# Pharmacological Modulation of Diacylglycerol-Sensitive TRPC3/6/7 Channels

**DOI:** 10.2174/138920111793937943

**Published:** 2011-01

**Authors:** Christian Harteneck, Maik Gollasch

**Affiliations:** 1 Institut für Pharmakologie & Toxikologie and Interfaculty Center of Pharmacogenomics and Pharmaceutical Research (ICePhA), Eberhard-Karls-Universität, Wilhelmstraße, Tübingen, Germany; 2 Charité University Medicine, Section Nephrology/Intensive Care, Campus Virchow, and Experimental and Clinical Research Center (ECRC), Augustenburger Platz 1, 13353 Berlin, Germany

**Keywords:** Diacylglycerol, hyperforin, ACA, SFKF-96365, calcium homeostasis.

## Abstract

Members of the classic type of transient receptor potential channels (TRPC) represent important molecules involved in hormonal signal transduction. TRPC3/6/7 channels are of particular interest as they are components of phospholipase C driven signalling pathways. Upon receptor-activation, G-protein-mediated stimulation of phospholipase C results in breakdown of phosphatidylinositides leading to increased intracellular diacylglycerol and inositol-trisphosphate levels. Diacylglycerol activates protein kinase C, but more interestingly diacylglycerol directly activates TRPC2/3/6/7 channels. Molecular cloning, expression and characterization of TRP channels enabled reassignment of traditional inhibitors of receptor-dependent calcium entry such as SKF-96365 and 2-APB as blockers of TRPC3/6/7 and several members of non-classic TRP channels. Furthermore, several enzyme inhibitors have also been identified as TRP channel blockers, such as ACA, a phospholipase A_2_ inhibitor, and W-7, a calmodulin antagonist. Finally, the naturally occurring secondary plant compound hyperforin has been identified as TRPC6-selective drug, providing an exciting proof of concept that it is possible to generate TRPC-selective channel modulators. The description of Pyr3 as the first TRPC3-selective inhibitor shows that not only nature but also man is able to generate TRP-selective modulators. The review sheds lights on the current knowledge and historical development of pharmacological modulators of TRPC3/6/7. Our analysis indicates that Pyr3 and hyperforin provide promising core structures for the development of new, selective and more potent modulators of TRPC3/6/7 activity.

## INTRODUCTION

With completion of the human genome project, more than 1200 G-protein coupled receptors have been identified ranging from well-known receptors to orphan receptors. Among them, a large group of receptors is activated by volatile compounds. The great diversity of ligands and receptors is integrated on the cellular level by a small number of cellular signalling cascades. The integration is mediated by G-proteins transducing ligand-dependent changes of receptor conformation into subsequent signalling pathways. In the context of calcium-permeable ion channels, hormonal signalling cascades integrated by the G-proteins of the G_q_ family and/or G-protein βγ-subunits are of particular interest. Their activation results in a phospholipase C-mediated breakdown of phosphatidylinositides leading to the formation of inositol 1,4,5-trisphosphate and diacylglycerol (Fig. (**1**)). Inositol 1,4,5-trisphosphate induces calcium release from intracellular stores, whereas diacylglycerol directly activates mammalian classic transient receptor potential (TRPC) channels (TRPC2, TRPC3, TRPC6 and TRPC7) in a protein kinase C-independent manner [1-3].

With respect to the fly origin of prototypic TRP channels, it is interesting to note that diacylglycerol is not able to directly stimulate the *Drosophila* TRPC members (TRP, TRPL, TRPγ). Instead, *Drosophila* TRPL and TRPγ are activated by phospholipase A_2_-dependent polyunsaturated fatty acids [4, 5]. The activation is directly caused by poly-unsaturated fatty acids like arachidonic acids (AA), but not downstream metabolites of AA which can be blocked by eicosatetraynoic acid. Eicosatetraynoic acid is routinely used as inhibitor of metabolic arachidonic acid pathways like lipoxygenases, cyclooxygenases and cytochrome P_450_ iso-enzymes.

In pharmacological research focussed on human diseases, TRPC2 in mostly ignored. TRPC2 is a pseudogene in human. Functional TRPC2 is found only in rodents, with TRPC2 involved in the pheromone signalling. Based on the broad expression profile of TRPC3 and TRPC6 being detected in many neuronal, epithelial and vascular smooth muscle cells [6], it is not surprising that both proteins are involved in a great variety of functions [7, 8]. In contrast, expression of TRPC7 is restricted to a few cell types and the physiological role of TRPC7 is still unclear [9]. This review will focus on pharmacological modulation of mammalian TRPC3/6/7. We will discuss a broad number of drugs that interfere with TRPC3/6/7 activity and function.

## INORGANIC BROAD RANGE TRP CHANNEL BLOCKERS

Since the first functional characterization of TRP channels, small molecules were introduced as tools for pharmacological modulation. For calcium-permeable ion channels, barium or strontium ions were initially used as divalent cations to study the selectivity and function of the new proteins [10, 11]. Barium entry measurements allowed to characterize heterologously expressed TRPC3 in DT40 and its contribution to receptor-dependent and independent signalling pathways [11]. On the other hand, TRPC6 were similarly characterized in vascular smooth muscle cells [10]. While divalent cations are able to permeate through the pores of non-selective TRPC3/6/7 cation channels, the trivalent cations gadolinium and lanthanum ions have been found to block TRPC3/6/7-mediated calcium entry [12-15]. The half-maximal concentration of lanthanum chloride necessary for TRPC3 inhibition was 4 µM, whereas more than 50 µM of lanthanum chloride was needed to block TRPC6 [12, 14]. These data prompted the usage of trivalent cations as tools to characterize TRPC channel-dependent signalling pathways in various cell types.

## ORGANIC BROAD RANGE TRP CHANNEL BLOCKERS

Organic synthetic blockers have been recognized to interfere with receptor-dependent and store-operated calcium entry mechanisms [16, 17]. SKF-96365, 1-{β[3-(4-methoxyphenyl)propoxy]-4-methoxyphenethyl}-1H-imidazole hydrochloride, (Fig. (**2**)) is an inhibitor of receptor-mediated as well as store-operated calcium entry mechanisms [16, 17]. Initially introduced as inhibitor of receptor-mediated calcium entry, SKF-96365 blocked ADP-induced calcium entry in platelets, neutrophils and endothelial cells with IC_50_ values of ~10 µM [18]. Usage of SKF-96365 allowed to discriminate between ATP- and bradykinin-induced calcium entry mechanisms in PC-12 cells and to characterize ATP- and N-formyl-L-methionyl-L-leucyl-L-phenylalanine (fMLP)-stimulated cation currents in HL-60 cells [19, 20]. Due to the initial characterization of SKF-96365 as blocker of receptor-induced calcium entry in mammals, attempts have been made to introduce SKF-96365 as selective blocker of diacylglycerol-regulated TRP channels, including TRPC3, TRPC6 [2, 12, 21]. Accordingly, Boulay *et al.* observed a complete block of heterologously expressed TRPC6 using 100 µM SKF-96365; the same concentration was effective in blocking endogenously expressed TRPC3 in human myometrial cells [12, 21].

A quite different history is obvious for 2-APB, 2-aminoethoxydiphenyl borate. Initially used as low cost alternative to the naturally occurring xestospongin C, 2-APB was introduced as blocker of inositol 1,4,5-trisphosphate receptors and used in this context for the characterization of store-operated calcium entry mechanisms [22]. In those early days, nearly all TRP channels have been characterized as store-operated calcium entry channels mostly based on data showing channel inhibition by 2-APB. However, it soon became clear that 2-APB targets more molecular structures than initially thought. Besides blocking all members of the TRPC and TRPM family [23-27], 2-APB stimulates TRPV-mediated calcium entry [28-30]. The discovery of stromal interactions molecules (STIM) (such as STIM1 and STIM2) and plasma membrane channels Orai1, Orai2 and Orai3 (also known as CRACM1, CRACM2 and CRACM3, respectively) has revealed novel molecular targets of 2-APB [31]. STIM1 and STIM2 represent the calcium sensors of the endoplasmic reticulum interacting and regulating Orai (or CRACM) channels. 2-APB interacts with CRACM channels; however, the interactions seem to be complex. CRACM1 and CRACM2 are blocked by 2-APB, whereas 2-APB activates a current in STIM1 and CRACM3 coexpressing cells [32-34].

Therefore, the molecular identification, cloning and functional characterisation of a great variety of ion channels enabled the identification of SKF-96365 and 2-APB as broad-range TRP (and CRACM) channel blockers. In contrast to them, other chemical structures initially described as specific inhibitors of enzymes and proteins have seen a revival as they were identified as TRP channel blockers. One of these molecules is N-(p-amylcinnamoyl) anthranilic acid (ACA). In order to interfere with the AA-dependent modulation of TRPM2, we characterized several compounds described as phospholipase A_2_ inhibitors. Together with arachidonyl trifluoromethyl ketone and p-bromophenacyl bromide, ACA was initially identified as a promising selective compound. However, subsequent detailed studies analyzed the complete blocking profile of ACA. It was found that ACA blocks TRPM2 and closely related TRPM8. ACA also inhibits TRPC3, TRPC6 and TRPV1 activity [35, 36].

Another example of a compound with a much broader spectrum profile than initially speculated is ML-9, 1-(5-Chloronaphthalene-1-sulfonyl)-1H-hexahydro-1,4-diazepine hydrochloride]. ML-9 has been initially identified as TRPC6 inhibitor [37]. In order to test whether TRPC6 activity is modulated by myosin light chain kinase (MLCK), Shi *et al*. tested several compounds described for inhibition of myosin light chain kinase [37]. Whereas MLCK-inhibiting peptides and other tools were found to be ineffective, Shi *et al*. demonstrated that ML-9 is as potent blocker of TRPC6. ML-9 has also been described as calmodulin-dependent MLCK inhibitor; it is structurally related to W-7, a compound described to interfere with calmodulin (Fig. (**2**)). In order to provide molecular insights into the pharmacology of TRPC6 channels, the effects of ML-9 and W-7 were studied on TRPC6 activated by hyperforin by FLIPR experiments Fig. (**3**). Using this approach, we tested several other structurally related compounds to get a first view on the structure-function relationships of TRPC6. Fig. (**3**) shows that all those compounds blocked hyperforin-induced TRPC6-mediated calcium entry with similar potencies. Curious to see whether W-7 is a TRPC6 or TRPC channel selective drug, we studied its inhibitory effect on TRPC6, TRPM2, TRPM3, TRPV4 and *Drosophila* TRPγ stimulated by either hyperforin, hydrogen peroxide, pregnenolone sulphate, 4α-phorbol-didecanoate or eicosatetraynoic acid, respectively (Fig. (**4**)). The IC_50_ values of TRPC6, TRPM2, TRPM3, TRPV4, *Drosophila* TRPγ inhibition were 28 µM, 26 µM, 15 µM, 65 µM, 5 µM, respectively. The data also show that ML-9 and related compounds may act as calmodulin-inhibitors in cell free experiments. However, in cell-based experiments it is unlikely that the compounds can cross the plasma membrane due to their sulphonamide structure providing good water solubility. Overall, these data demonstrate that many “established” pharmacological tools in TRP channel research have to be critically re-evaluated to draw correct conclusions about the identity of TRP channels involved in biological processes and the pharmacology of the channels.

## SELECTIVE MODULATORS OF TRPC3/6/7 CHANNELS

The discovery of hyperforin as selective TRPC6 channel modulator is exciting by the fact that modulation of TRP channels by secondary plant compounds is not restricted to TRP channels involved in thermo sensation and pain [38]. The discovery of hyperforin as selective TRPC6 channel modulator was initially surprising. Since hyperforin is the first naturally occurring modulator of channels of the TRPC family, there is hope that further TRPC subfamily modulators can be discovered. Hyperforin, a bicyclic polyprenylated acylphloroglucinol derivate, is the main active ingredient of St. John´s wort extract, which is widely used as the herbal alternative to treat depression. In 2002, 12% of US adults reported to use St. John’s wort extract within the last 12 months [39]. Several recent clinical trials [40-42] and Cochrane meta analysis confirm the clinical efficacy and good tolerability of St. John’s wort for depression [43]. The effect of classic, synthetic antidepressants is based on inhibition of neuronal uptake of serotonin, dopamine and norepinephrine by a direct block of the transport proteins (Fig. (**5**)). The neuronal uptake of serotonin, dopamine and noradrenaline by neurotransmitter transporters are frequently dependent on electrochemical gradients, such as Na^+^ gradients, across the plasma membrane. In contrast to classic, synthetic anti-depressants, hyperforin reduces monoamine uptake by elevating the intracellular sodium concentration and subsequent elimination of the sodium gradient as required for neurotransmitter transporter function [44, 45]. The effect is mediated by direct activation of TRPC6 [38]. By direct hyperforin-channel interaction, sodium and calcium permeate the pore of TRPC6 see Fig. (**5**). Increase in intracellular sodium counterbalances the sodium gradient across the plasma membrane, thereby, indirectly inhibiting neurotransmitter transporters. The acute increase in neurotransmitter in the synaptic cleft is followed by calcium-dependent neuronal differentiation see Fig. (**5**). Hyperforin likely integrates inhibition of neurotransmitter uptake and neurotrophic property by specific activation of TRPC6.

In traditional herbal medicine, St. John´s wort is used for treatment of depression [43] and can be used for topical treatment of superficial wounds, burns and dermatitis [46]. The skin barrier is formed by keratinocytes. During their passive transport to the skin surface, change in morphology and expression profile is indicative for keratinocyte differentiation, until they finally scale up in danders. In cell culture studies, keratinocytes differentiation can be induced by increasing extracellular calcium concentration leading to activation of the calcium receptor (CaR). In addition, increases in extracellular calcium concentration enhance the calcium gradient across the plasma membrane resulting passively in increased intracellular calcium concentration. Several publications have described endogenous TRPC expression profiles in keratinocytes. Cai *et al.* detected TRPC1, TRPC5, TRPC6 and TRPC7 in gingival keratinocytes [47], whereas Beck *et al*. showed expression of TRPC1, TRPC4, TRPC5 and TRPC7 in HaCaT keratinocytes [48]. TRPC1 as well as TRPC4 have been implicated in the CaR triggered elevation of [Ca^2+^]_i_ [47, 48]. Furthermore, the expression profile of TRPC channels is dynamically regulated. Calcium-induced differentiation of gingival keratinocytes results in elevated expression of TRPC1, TRPC5, TRPC6 and TRPC7 [47]. Treatment of keratinocytes with betulin, a triterpene extracted from the outer bark of birch (betulae cortex), results in increased TRPC6 expression [49]. In agreement with these data, we recently showed that TRPC6 is functionally expressed in keratinocytes and contributes to differentiation processes. Accordingly, inactivation of TRPC6 translation decreases calcium-dependent differentiation monitored by keratin 1 and keratin 10 expression [50]. Furthermore, the experimental set up allowed us to show that TRPC6 represents the molecular target for triggering differentiation processes in keratinocytes [50]. In summary, TRPC6 seems to serve as the molecular target for hyperforin-induced neuronal and keratinocyte differentiation.

In clinical practice, the use of hyperforin is handicapped by its capacity to induce the cytochrome P450 (CYP) system leading to increased expression of CYP3A and CYP2C [51, 52]. The induction of CYP3A and CYP2C is critical as these enzymes metabolise up to 50% and 20% of several therapeutically used drugs, respectively. In accordance, hyperforin affects the metabolism and corresponding plasma concentrations of at least 70% of the therapeutically used drugs [51, 52]. On the other hand, CYP2C enzymes are important in the generation of biologically active molecules such as epoxyeicosatrienoic acids (EETs) and hydroxyeicosatrienoic acids (HEETs), which originate from arachidonic acid in both liver and extrahepatic tissues such as kidney, heart, aorta, and blood vessels [51]. EETs regulate vasodilation [53] which is of potential interest as to understand why in patients treated with St. John´s wort extracts systemic blood pressure and heart rate seem to be unchanged [54]. The stable blood pressure is amazing as TRPC6 is expressed in vascular smooth muscle cells and obviously involved in regulation of vascular tone [10, 55-58]. From this data, one would expect that under St. John´s wort therapy systemic blood pressure would rise due to increased arterial blood vessel constriction induced by increased TRPC6-mediated calcium entry. This may occur shortly after beginning of therapy, whereas in steady state phase this effect might be compensated by increased EET production via CYP2C induction. Block of voltage-gated calcium channels may play an additional role [59, 60]. The long-term effects of hyperforin therapy on kidney function are unclear since gain-of-function mutations of TRPC6 have been implicated in familial focal segmental glomerulosclerosis [61-64]. However, the mechanism of this effect is rather unclear since not all mutations showed increased channel function.

Hyperforin is a polyprenylated bicyclic acylphloroglucinol derivative. Extensive chemical degradation and derivatisation studies have established its cage like structure (Beerhues, 2006). Exposed to light and oxygen, hyperforin is very unstable, which may additionally contributes to differences in hyperforin content in St. John´s wort extract leading to the comment “*The association of country of origin and precision with effects sizes complicates the interpretation*.” in the Cochrane report [43]. Based on the core structure of hyperforin, the phloroglucinol structure, we characterized several phloroglucinol derivatives [65]. Symmetric 2,4-diacylphloroglucinol derivative have been identified as active compounds stimulating heterologously as well as endogenously expressed TRPC6 [65]. Despite massive simplification and reduction in complexity, the symmetric 2,4-diacylphloroglucinol derivatives showed a comparable activity profile tested in several test systems such as transmitter uptake, neurotrophic assay, and TRPC6 activity. Notably, chemical modification of structures provided by nature generally result in loss of selectivity and activity. However, in the case of hyperforin, the symmetric 2,4-diacylphloroglucinol derivatives preserved their potency in activation of TRPC6 channels, whereas closely related TRPC3 and TRPC7 channels remained unaffected by these drugs [65].

The structure of the symmetric 2,4-diacylphloroglucinol derivatives (Hyp derivatives) is also interesting in the context of other TRPC-selective pharmacological tools (Fig. (**6**)). Recently, Kiyonaka *et al*. described Pyr3 as selective TRPC3 inhibitor [66]. The compound Pyr3 is based on the precursor BTP2, described as inhibitor of store-operated calcium release-activated calcium (CRAC) channels essential for T-cell activation and proliferation [67]. In contrast to BTP2, Pyr3 is a selective inhibitor of TRPC3 channels leaving the activity of other TRPC channel members unchanged [66]. For cross linking experiments, a Pyr3 derivative, Pyr-PP, was developed. Pyr-PP was shown to be activated and chemically linked to TRPC3 by UV-light activation [66].

In summary, beside several pharmacological tools, which can be classified as broad-range TRP channel blocker, Pyr3, hyperforin and the Hyp derivatives represent the first TRPC-selective modulators. While Pyr3 selectively blocks TRPC3 channels, hyperforin and the Hyp-derivative stimulate TRPC6 channels. Both core structures provide the basis for further development of new, selective and more potent modulators of TRPC3/6/7 activity.

## Figures and Tables

**Fig. (1):**
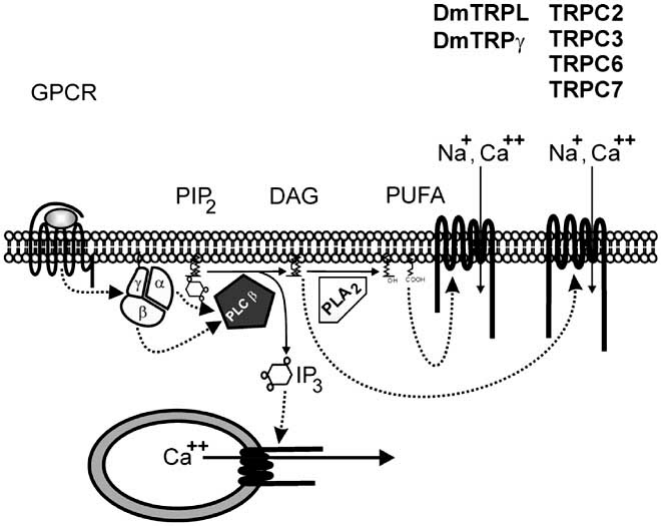
Signalling cascade leading to TRPC3/6/7 activation. Hormonal stimulation of G-protein coupled receptor (GPCR) activates phospholipase C isoforms (PLC β) via a G-protein dependent mechanism. Phospholipase C activity results in the breakdown of phosphatidylinositides (PIP_2_) leading to the formation of inositol 1,4,5-trisphosphate (IP_3_) and diacylglycerol (DAG). Inositol 1,4,5-trisphosphate as ligand of inositol 1,4,5-trisphosphate receptors located at the endoplasmic reticulum induce calcium release from intracellular stores, whereas DAG activates mammalian TRPC2, TRPC3, TRPC6 and TRPC7 channels. In contrast to the mammalian signalling cascade, *Drosophila* TRPL and TRPγ are activated by poly-unsaturated fatty acids (PUFA) generated by phospholipase A_2_ (PLA_2_) from subsequent degradation of diacylglycerols.

**Fig. (2):**
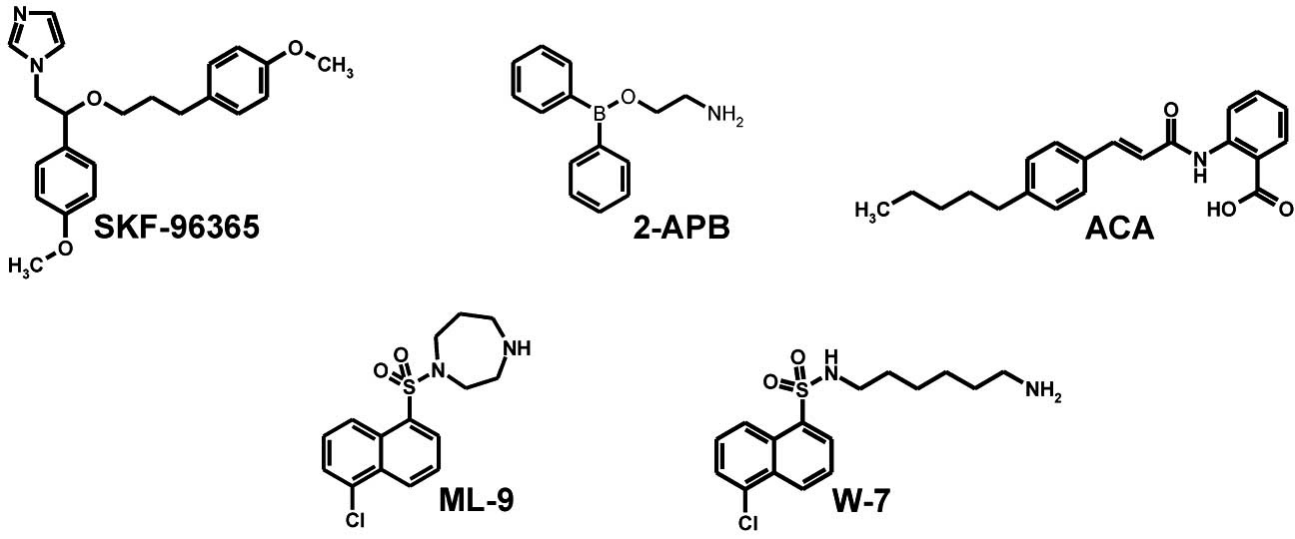
Chemical structures of broad range TRP channel blocker. SKF-96365: 1-{β[3-(4-methoxyphenyl)propoxy]-4-methoxyphenethyl}-1H-imidazole hydrochloride; 2-APB: 2-aminoethoxydiphenyl borate; ACA: N-(p-amylcinnamoyl)anthranilic acid; ML-9: [1-(5-Chloronaphthalene-1-sulfonyl)-1H-hexahydro-1,4-diazepine hydrochloride]; W-7: N-(6-Aminohexyl)-5-chloro-1-naphthalenesulfonamide hydrochloride.

**Fig. (3):**
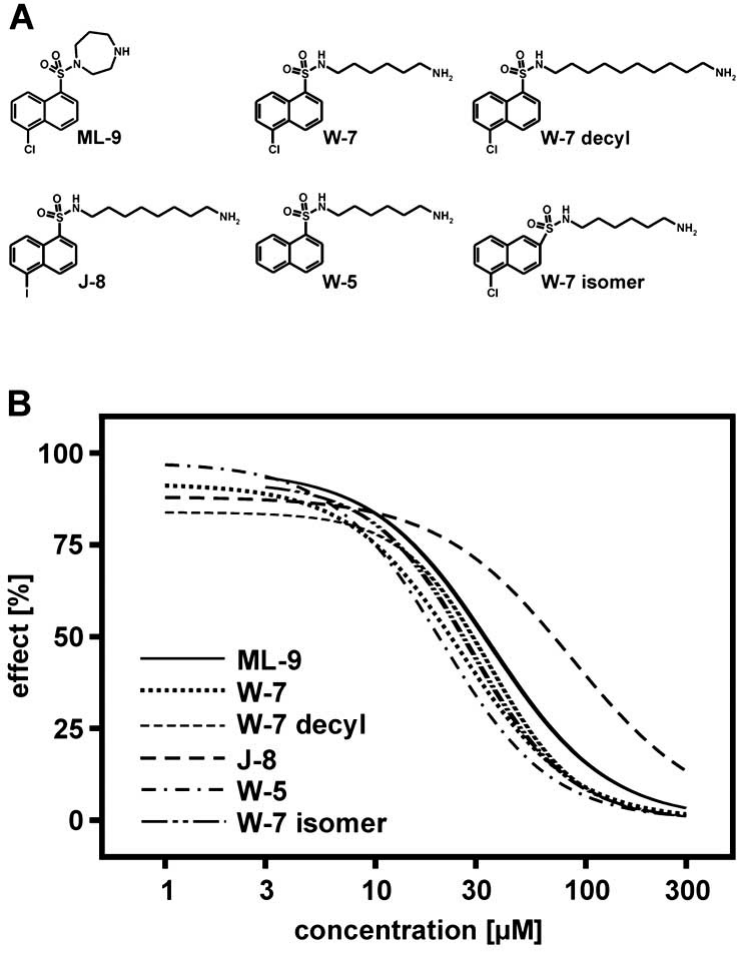
Concentration-response relationship of ML-9 and W-7 related compounds on hyperforin-induced, TRPC6-mediated fluorescence changes. **A**) The chemical structures of the tested compound is shown. ML-9: 1-(5-Chloronaphthalene-1-sulfonyl)-1H-hexahydro-1,4-diazepine; W-7: N-(6-Aminohexyl)-5-chloro-1-naphthalenesulfonamide; W-5: N-(6-Aminohexyl)-1-naphthalenesulfonamide; W-7 isomer: N-(6-Aminohexyl)-5-chloro-2-naphthalenesulfonamide; J-8: (N-8-Aminooctyl)-5-iodo-1-naphthalenesulfonamide; W-7 decyl: N-(6-Aminodecyl)-5-chloro-1-naphthalenesulphonamide. **B**) Data from a representative experiment show the effect of ML-9, W-7, W-7 decyl, W-7 isomer, W-5 and J-8 on calcium entry in TRPC6-expressing cells upon hyperforin stimulation. Calcium entry was measured using FLIPR^Tetra^ and data analysed as described in Jörs *et al*. [5]. The data were calculated from one experiment of at least three experiments performed in quadruplicates per concentration and TRP channel. The concentration-response curves determined by calcium imaging showed a quite distinct inhibition profile. The IC_50_ values of ML-9, W-7, W-7 isomer, W-7 decyl W-5, J-8 were 36 µM, 26 µM, 35 µM, 29 µM, 20 µM, 85 µM, respectively.

**Fig. (4):**
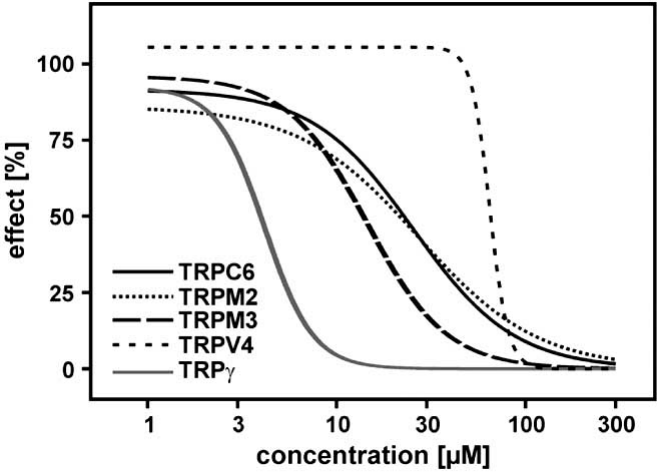
Concentration-dependent inhibition of TRPC6-, TRPM2-, TRPM3-, TRPV4- and TRPγ-mediated calcium entry by W-7. Fluorescence of TRPC6-, TRPM2-, TRPM3-, TRPV4- and TRPγ-expressing, fluo-4-loaded cells in were stimulated with hyperforin (10 µM), hydrogen peroxide (5 mM), pregnenolone sulphate (35 µM), 4α-phorbol-didecanoate (5 µM) and eicosatretranoic acid (20 µM), respectively. Calcium entry was measured using FLIPR^Tetra^ and data analysed as described in Jörs *et al*. [5]. The data were calculated from one experiment of at least three experiments performed in quadruplicates per concentration and TRP channel. The concentration-response curves determined by calcium imaging showed a quite distinct inhibition profile. The IC_50_ values to block TRPC6, TRPM2, TRPM3, TRPV4, *Drosophila* TRPγ were 28 µM, 26 µM, 15 µM, 65 µM, 5 µM, respectively.

**Fig. (5):**
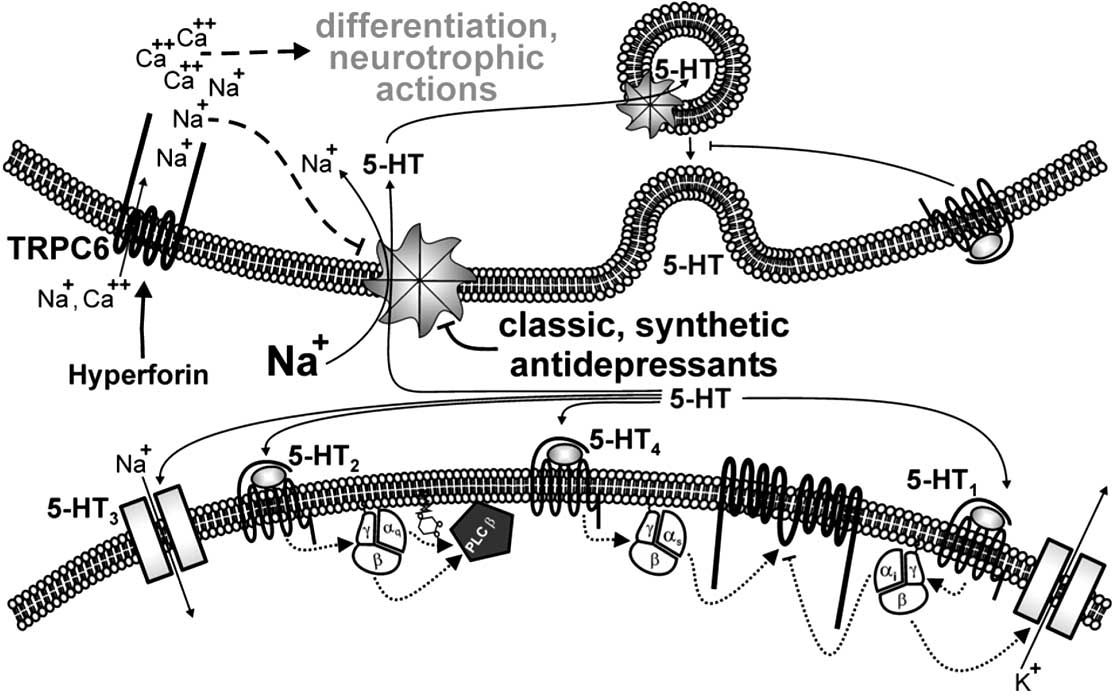
Mechanistic model of the hyperforin-dependent antidepressive effect. Within the neurotransmitter circuit of release and uptake, hyperforin and classic, synthetic antidepressants attack different targets resulting in reduced presysnaptic uptake of neurotransmitter and increase concentration of neurotransmitter in the synaptic cleft. Whereas classic synthetic antidepressants directly block the neurotransmitter transporters, hyperforin activates TRPC6 channel in proximity of the neurotransmitter transporters. TRPC6 is a non-selective cation channel enabling the permeation of sodium and calcium. Sodium entry mediated by TRPC6 activation reduced the sodium gradient across the plasma membrane. As the activity of the neurotransmitter transporters depends on the electrochemical sodium gradient, TRPC6 activity indirectly inhibits neurotransmitter uptake. On the other hand, TRPC6-mediated calcium entry may trigger calcium-dependent differentiation processes responsible for changes in neuronal plasticity.

**Fig. (6):**
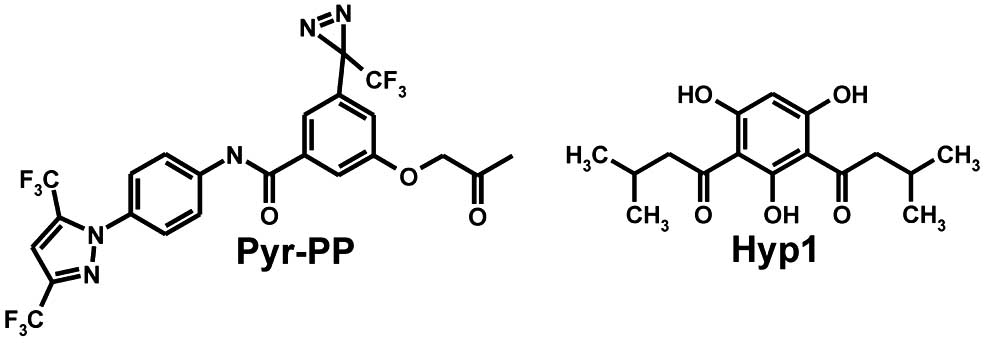
Comparison of the chemical structure of Hyp1 and Pyr-PP.
